# How does the digital economy enhance carbon emission efficiency in the logistics industry? Empirical evidence from 30 Chinese provinces

**DOI:** 10.1038/s41598-025-20485-w

**Published:** 2025-10-21

**Authors:** Xiaoxuan Xie, Kyoungsuk Choi, Xina Ji

**Affiliations:** 1 Department of International Trade, Jeonbuk National University, Jeonju-si, Jeollabuk-do Republic of Korea; 2 College of International Economics and Trade, Ningbo University of Finance and Economics, Ningbo, China

**Keywords:** Digital economy, Carbon emission efficiency, Logistics industry, Industrial structure upgrading, Energy structure, Spillover effect, Climate sciences, Environmental social sciences

## Abstract

The rapid advancement of digital infrastructure has accelerated the rise of the digital economy, now a key driver of industrial transformation. This study investigates how the digital economy influences carbon emission efficiency in the logistics industry, drawing on panel data from 30 Chinese provinces between 2012 and 2021. It further examines the mediating roles of industrial structural upgrading and energy structure, as well as heterogeneity and spatial spillover effects. The digital economy was measured using the entropy method, while carbon emission efficiency was assessed with the super-SBM model, followed by benchmark regression to test inter-factor relationships. The findings reveal a nonlinear relationship between the digital economy and carbon emission efficiency. Specifically, the digital economy enhances efficiency both directly and indirectly by fostering industrial structure upgrading and optimizing energy structure. Significant regional disparities were also identified, with nonlinear spatial spillover effects on neighboring provinces. Unlike prior research, this study employs a more rigorous methodological framework with a focused analysis of the logistics industry. The results enrich academic understanding of the digital economy–carbon emission efficiency nexus and offer practical insights into leveraging digital transformation to promote sustainable and green development in the logistics industry.

## Introduction

 The escalating severity of greenhouse gas emissions underscores the growing importance of achieving carbon neutrality and net-zero targets^1–7^. Carbon neutrality is not merely an environmental concept but a practical commitment that extends across industries—particularly the logistics industry—where improving carbon emission efficiency (CEE) has become a critical pathway to sustainable development^8^. As a high-energy-consuming and high-emission industry, the logistics industry therefore faces pressing demands for green transformation^9^. According to the China Emission Accounts and Datasets (CEADS), China’s total carbon emissions across all industrial sectors reached 10,356.265 Mt in 2021, with the logistics industry ranking fourth, making it one of the major sources of carbon emissions^10^. In recent years, driven by the rapid expansion of e-commerce and related industries, carbon emissions from the logistics industry have shown a significant upward trend^11^. Therefore, for the sustained prosperity of the logistics industry, reducing carbon emissions by enhancing CEE has become an important new challenge for China.

Against this background, identifying new drivers to promote low-carbon logistics has become imperative. With the deep integration of economic growth and digitalization, the digital economy (DE) has gradually emerged as a crucial force in sustaining economic stability and facilitating energy conservation and emission reduction^12,13^. In line with this trend, the Chinese government introduced the “14th Five-Year Plan,” which emphasizes strengthening digital industrialization and promoting the comprehensive digital transformation of industries^14–16^. Consequently, as digital technologies increasingly penetrate sustainable development practices, the nexus between the DE and carbon emissions has attracted growing academic attention^7,17^. For instance, Xie and Zhang (2022), using provincial data from China for 2003–2018, found that although the industrial sector exhibited relatively low CEE overall, the development of the DE significantly enhanced its efficiency, with a stage-dependent threshold effect^18^. Similarly, Wang et al. (2024), based on panel data from 29 Chinese provinces for 2011–2020, revealed an inverted U-shaped nonlinear relationship between the DE and tourism-related carbon emissions, with a turning point at 0.698^19^. In addition, Wu et al. (2024) examined the emission-reduction pathways of digital technologies in the construction industry, showing that their effects were realized through fostering green innovation and technological progress^20^. Zhang et al. (2025), employing a spatial Durbin model, investigated coastal fisheries and demonstrated that the DE not only reduced carbon emissions locally but also generated spatial spillover effects on neighboring regions^21^. Furthermore, Li et al. (2025) provided additional evidence on the nonlinear impacts of the DE on industrial CEE as well as its spatial transmission mechanisms^22^.

Existing research has predominantly concentrated on traditional sectors such as manufacturing, construction, and fisheries, with limited attention to the logistics industry, which is undergoing rapid digital transformation^23^. Moreover, prior studies have emphasized mainly carbon emission reduction rather than improvements in CEE. These gaps highlight the need for more systematic investigation into how DE influences CEE in the logistics industry.

To address this gap, the present study investigates the relationship between the DE and CEE, with a specific focus on China’s logistics industry. Four key research questions are examined: (1) Does the DE exert a nonlinear effect on the CEE of the logistics industry? (2) Does the DE indirectly influence logistics CEE through industrial structural upgrading (ISU) and energy structure (ES) adjustments? (3) Does the development of the DE generate spatial spillover effects on the CEE of logistics industries in neighboring regions? and (4) Does the impact of the DE on logistics CEE vary across regions and levels of economic development?

To answer these questions, this study applies a rigorous methodological framework based on panel data from 30 provinces during 2012–2021. We first constructed a DE index using the entropy method and measured CEE in the Chinese logistics industry through the super-SBM model. Building on this, we employed a two-way fixed-effects model, a mediation-effects model, and a spatial Durbin model (SDM) to systematically analyze the mechanisms and spatial spillover effects of the DE on logistics CEE.

**Fig. 1:**
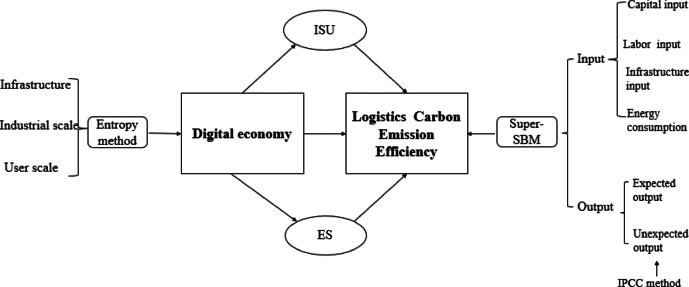
Research framework.

This study contributes to literature in three ways. First, it extends existing research by focusing on the logistics industry, a sector that is rapidly undergoing digital transformation but has received limited scholarly attention. Second, it shifts the analytical lens from carbon emission reduction to CEE, thereby providing a more nuanced understanding of the DE’s role in promoting sustainable development. Third, by integrating nonlinear effects, mediating mechanisms (ISU and ES), and spatial spillover effects into a unified framework, this study offers both methodological rigor and practical insights for advancing low-carbon logistics (Fig. 1).

## Literature review and research hypotheses

### The relationship between the digital economy and carbon emission efficiency

The DE is expanding rapidly with the advancement of Fourth Industrial Revolution technologies, particularly information and communication technology (ICT) and digital infrastructure. Accordingly, DE has gained increasing attention in academia, with scholars examining its implications for CEE across different countries and industries. From an industrial perspective, Xie et al. (2022) found that DE contributes to improving CEE in China’s manufacturing industry¹³, while Dong et al. (2025) confirmed that rural DE development significantly enhances agricultural net carbon efficiency²⁵. Feng et al. (2025) demonstrated a nonlinear relationship between DE and CEE in the agricultural industry²⁴, and other studies have likewise confirmed similar nonlinear effects across various industries²⁶. From a regional perspective, Zhang et al. (2024) analyzed the impact of DE on low-carbon logistics efficiency in RCEP countries and identified a significant positive spatial correlation. Their findings indicate that the DE enhances low-carbon logistics efficiency not only domestically but also in neighboring countries through spatial spillover effects²⁷. Similarly, Xia et al. (2025), based on city-level data in China, argued that the DE can effectively enhance CEE^17^. Du et al.(2025), focusing on the Beijing–Tianjin–Hebei, Yangtze River Delta, and Pearl River Delta urban agglomerations, provided evidence of a nonlinear relationship between the DE and CEE^24^. Another study using panel data from 30 Chinese provinces further confirmed that the DE significantly improves CEE while generating spatial spillover effects on adjacent regions^25^.

A review of the literature indicates that previous studies examining the relationship between DE and CEE have primarily focused on two themes: facilitation effects and nonlinear dynamics. Studies emphasizing the facilitation effect suggest that the DE enhances CEE by leveraging data and information technologies to improve factor mobility and promote industrial digitalization. By contrast, studies adopting a nonlinear perspective highlight the existence of an inverted U-shaped relationship between the two factors. Specifically, while digital advancements may initially increase carbon emissions, once the DE reaches a certain threshold, its impact shifts from promoting emissions to enhancing efficiency. Accordingly, we infer that in the logistics industry, the DE initially contributes to improvements in CEE but, beyond a certain threshold, begins to hinder efficiency, thereby forming an inverted U-shaped relationship. Building on these insights, we propose the following hypotheses to test the nonlinear effect of the DE on CEE in the logistics industry, a sector that has received limited attention in prior research.

#### H1

The DE has a nonlinear effect on the CEE of the logistics industry.

### Industrial structural upgrading and energy structure as mediator

Existing studies have highlighted that industrial structure upgrading (ISU) plays a critical role in the process through which the DE influences carbon emissions^26^. As its core, this process lies in the integration of industrial technological progress and economic structural optimization. Such integration not only facilitates the expansion of industries into high-end segments within the vertical division of labor but also promotes the coordinated development of related industries within the horizontal division of labor. In this way, the DE and industrial structure mutually reinforce each other and jointly foster low-carbon transformation^27^. Wu and Shao (2022)empirically confirmed this perspective, demonstrating that the DE significantly promotes ISU^28^. Similarly, using provincial panel data for China from 2010 to 2019, Chang et al. (2023) found that the DE suppresses carbon emission intensity, with ISU serving as a key mediating factor^27^. Lu et al. further argued that innovation-driven industrial clusters can effectively enhance urban CEE, with ISU again functioning as an important mediator^29^. In addition to ISU, the DE indirectly affects carbon emissions through the energy structure (ES). ES refers to the proportion of coal consumption in total energy consumption. Among various types of energy consumption, coal is the energy resource with the highest carbon dioxide emissions. By optimizing energy production and consumption patterns, accelerating the development and application of renewable energy, and advancing the low-carbon transformation of the energy system, the DE can improve CEE. Previous studies have also shown that the ES operates as a mediating mechanism in the relationship between the DE and carbon emissions^30^. Based on the literature, the following hypotheses are proposed.

#### H2

The DE has a positive effect on the CEE of the logistics industry through the mediating role of ISU.

#### H3

The DE has a positive effect on the CEE of the logistics industry through the mediating role of the ES.

### Spatial spillover effects of the digital economy on carbon emission efficiency

According to spatial econometric theory and regional innovation theory (Feldman & Audretsch, 1999), the development of one region’s economy often generates spillover effects on adjacent areas, particularly in the domains of technological innovation and environmental performance^31^. The DE, characterized by openness, cross-spatial connectivity, and shared economic activities, has the inherent ability to transcend geographical constraints and reduce information asymmetry through highly efficient digital platforms. These features substantially diminish spatial barriers among market participants and enhance the intensity and reach of interregional economic interactions^32^. By leveraging its boundary-transcending nature, the DE not only improves CEE within a given region but also exerts positive externalities on neighboring regions. Prior studies have confirmed that digital technologies can lead to significant spatial spillover effects, particularly in terms of environmental outcomes^32,33^. Furthermore, recent research indicates that the spatial spillover effects of the DE on surrounding regions may also follow a nonlinear pattern, often exhibiting an inverted U-shaped relationship^34^. Based on this analysis, we propose the following hypothesis:

#### H4

The DE exerts a nonlinear spatial spillover effect on the CEE of the logistics industry in neighboring regions.

## Methodology and data

### Methodology

#### Baseline regression model

To investigate the impact of the DE on logistics industry’s CEE, we first construct a baseline regression model with two-way fixed effects. By incorporating both individual and time fixed effects^35^, the model controls for unobservable individual characteristics and time-invariant factors, thereby reducing omitted variable bias and improving the robustness of the estimation results. The model is specified as follows:

$$CEE_{it} = \varphi_{0} + \varphi_{1}DE_{it} + \varphi_{2}DE_{it}^{2} + \varphi_{3}C_{it} + \mu_{i} + \gamma_{t} + \varepsilon_{it}$$

where *i* denotes the province, *t* denotes the year, and CEE_it_ represents the CEE of the logistics industry in province *i* at time *t*. C_*it*_ is a vector of control variables. $\:{\mu\:}_{i}$ is the provincial fixed effect, $\:{\gamma\:}_{t}\:$is the time fixed effect, and $\:{\epsilon\:}_{it}$is the random error term.

### Mediation effect model

In addition to the direct impact of the DE on CEE, as discussed in the previous section, the DE may influence CEE by M(mediating variable). To examine this theoretical mechanism, the following model was constructed:

$$M_{it} = \beta_{0} + \beta_{1}DE_{it} + \beta_{2}DE_{it}^{2} + \beta_{3}C_{it} + \mu_{i} + \gamma_{t} + \varepsilon_{it}$$

$$CEE_{it} = \omega_{0} + \omega_{1}DE_{it} + \omega_{2}DE_{it}^{2} + \omega_{3}M_{it} + \omega_{4}C_{it} + \mu_{i} + \gamma_{t} + \varepsilon_{it}$$

### SDM model

To test the proposed hypotheses, this study introduces a nonlinear SDM. This model is particularly effective in capturing the spatial heterogeneity between the DE and CEE, and in revealing the differentiated impact of DE development on CEE across regions^36^. Compared with traditional models, the SDM not only characterizes the direct effect of local DE development on CEE but also accounts for spatial spillover effects from neighboring regions. This allows for a more comprehensive investigation of the interaction mechanisms among regions^37^. The specific model is specified as follows:

$$\begin{aligned}CEE_{it} &= \rho_{1} \sum_{j=1}^{n} W_{ij} CEE_{jt} + \alpha_{1} DE_{it} + \alpha_{2} DE_{it}^{2} + \theta_{1} \sum_{j=1}^{n} W_{ij} DE_{it} \\&\quad + \theta_{2} \sum_{j=1}^{n} W_{ij} DE_{it}^{2} + \delta C_{it} + \nu \sum_{j=1}^{n} W_{ij} C_{it} + \mu_{i} + \gamma_{t} + \varepsilon_{it}\end{aligned}$$

$\:{C}_{it}$denotes the control variables, while $\:\:{\alpha\:}_{1}$and $\:{\alpha\:}_{2}$represent the coefficients of $\:DE\:$and $\:DE^2\:$respectively.

$\:{\rho\:}_{1}$, $\:{\theta\:}_{1}$, $\:{\theta\:}_{2}$and $\:\nu\:$are the spatial lag coefficients.

### Data

#### Dependent variable and measurement: CEE

CEE of the logistics industry was measured using the Super-SBM model. Based on previous studies, the following variables were selected for CEE analysis. The input factors include logistics capital input, logistics industry employees, logistics resource consumption, and logistics infrastructure. The value added by the logistics industry is considered the expected output variable, while carbon emissions from the logistics industry are regarded as unexpected output variables. Table 1 presents the variables employed in the study.

**Table 1:** Input and output variables for constructing the logistics industry’s CEE.

Indicator category	Indicator name	Indicator description	Unit	Data source
Input	Capital stock	Investment in fixed assets in the logistics industry (Dai et al., 2025^38^)	100 million yuan	China Statistical Yearbook
	Number of staffs	Logistics labor inputs (Dai et al., 2025^38^)	10,000 people	
	Energy consumption	Logistics’ energy consumption (Yao et al., 2024^37^)	10,000 tons of standard coal	China Energy Statistical Yearbook
	Infrastructure	Total comprehensive mileage of transportation line (Ye et al., 2022^39^)	km	China Statistical Yearbook
Expected output	Value added to the logistics industry	Annual increase in output value of the logistics industry (Li et al., 2025^40^)	100 million yuan	
Unexpected outputs	CO_2_ emissions	Carbon emissions of logistics industry calculated using the IPCC method(Yao et al., 2024^37^)	10,000 tons	China Energy Statistical Yearbook

Traditional DEA models, such as the CCR and BCC, are radial in nature and have been widely applied for efficiency evaluation. However, they tend to overestimate efficiency when input redundancy or output deficiency exists and may not simultaneously capture multiple input-output dimensions effectively. To overcome these limitations, this study adopts the Super-SBM model proposed by Tone^41^, a non-radial DEA approach that incorporates unexpected outputs and overcomes the restriction of efficiency values being capped at 1. Compared with conventional DEA models, the Super-SBM provides a more comprehensive and precise efficiency assessment by accounting for both input excess and output shortfall. Accordingly, it was selected to measure CEE in this study. Assume that there are $\:{DMU}_{j}$ (j = 1,2,3,.,30), each with *m* inputs$\:{\:X}_{i}$($\:{x}_{1k}$, $\:{x}_{2k}$,., $\:{x}_{mk}$), *s* expected outputs Yr ($\:{y}_{1k}$, $\:{y}_{2k}$,., $\:{y}_{sk}$), and *u* unexpected outputs $\:{O}_{q}$ ($\:{o}_{1k}$,, $\:{o}_{2k}$,., $\:{o}_{uk}$). The specific calculation process is as follows.

$$\:\begin{array}{c}minp=\frac{\:\:1+\frac{1}{m}\sum\:_{i=1}^{m}\frac{{{s}_{i}}^{-}}{\:\:{x}_{ik}}}{1-\frac{1}{s+u}\left(\sum\:_{r=1}^{s}\frac{{s}_{r}^{+}}{{y}_{rk}}+\sum\:_{q=1}^{u}\frac{{s}_{q}^{o-}}{{o}_{qk}}\right)} \end{array}$$

subject to

$$\:\sum\:_{\begin{array}{c}j=1\\\:j\ne\:k\end{array}}^{n}{\lambda\:}_{j}{x}_{ij}-{s}_{i\:\:\:\:}^{-}\le\:{x}_{ik,\:\:\:\:\:\:\:\:\:\:\:\:\:\:\:\:\:\:\:\:\:\:\:\:\:\:\:\:\:\:\:\:}i=\text{1,2},,\dots\:\dots\:m$$

$$\:\sum\:_{\begin{array}{c}j=1\\\:j\ne\:k\end{array}}^{n}{\lambda\:}_{j}{y}_{rj}+{s}_{r\:\:\:\:}^{+}\ge\:{y}_{rk,\:\:\:\:\:\:\:\:\:\:\:\:\:\:\:\:\:\:\:\:\:\:\:\:\:}\:\:\:\:\:r=\text{1,2},,\dots\:\dots\:s$$

$$\:\:\:\:\sum\:_{\begin{array}{c}j=1\\\:j\ne\:k\end{array}}^{n}{\lambda\:}_{j}{o}_{qj}-{s}_{q\:\:\:\:}^{o-}\le\:{o}_{qk,\:\:\:\:\:\:\:\:\:\:\:\:\:\:\:\:\:\:\:\:\:\:\:\:\:\:\:\:\:\:\:}q=\text{1,2},,\dots\:\dots\:u$$

$$\:1-\frac{1}{s+u}\left(\sum\:_{r=1}^{s}\frac{{s}_{r}^{+}}{{y}_{rk}}+\sum\:_{q=1}^{u}\frac{{s}_{q}^{o-}}{{o}_{qk}}\right)>0$$

$$\:{\lambda\:}_{j,\:\:}{\:s}^{-\:\:},\:{s}^{+}\ge\:0$$

$$\:j=\text{1,2},\dots\:,n\:(j\ne\:k)$$.

Here, $\:p$ denotes the CEE of the logistics industry in each province. when $\:p\ge\:1$ the logistics industry’s CEE in that province is considered relatively efficient, whereas when $\:p<\:1$, the CEE is regarded as relatively ineffect. $\:{s}_{i}^{-}$;$\:{s}_{r}^{+}$and $\:{s}_{q}^{o-}$ are the slack variables of inputs, expected outputs, and unexpected outputs respectively. $\:{x}_{ij\:}$is the *i*-th inputs of $\:{DMU}_{j}$; $\:{y}_{rj}$is the *r*-th expected output of $\:{DMU}_{j}$; $\:{o}_{qj}$is the *q*-th unexpected output of $\:{DMU}_{j}$; $\:{\lambda\:}_{j}$represents the weight of the $\:{DMU}_{j}$.

#### Independent variable and measurement: DE

There are no universally accepted standards in academia for evaluating the development of DE. Following Yi et al. (2022), this study evaluates the DE using three key dimensions: infrastructure, industry, and users (see Table 2)^32^. To minimize potential biases arising from subjective judgment, the entropy method was employed to construct the DE index.

**Table 2:** Dimensions and variables for the DE Index.

Variables	Details (unit)	Attribute
Infrastructure	Fiber optic cable line length (10,000 km)	+
	Mobile phone exchange capacity (10,000 households)	+
	Internet broadband access ports (10,000)	+
Industrial scale	Total telecommunication services (100 million yuan)	+
	Revenue from main business of telecommunication industry (100 million yuan)	+
	Revenue from software operations (100 million yuan)	+
	Employed people engaged in information transmission, software and information technology services in urban units (10,000 persons)	+
User scale	Internet broadband access subscribers (10,000 households)	+
	Digital television user (10,000 households)	+
	Number of web pages (10,000)	+
	Number of domains (10,000)	+
	Mobile phone penetration (department/hundred people)	+
	Mobile subscribers at year-end (10,000)	+

In this study, the entropy method is employed to measure the DE index. The entropy method, recognized for its objectivity, assigns weights to indicators by utilizing the inherent information in the data, thereby minimizing subjective judgment in the weighting process. This approach ensured that the relative importance of each indicator is accurately reflected. The specific calculation steps are as follows.

First, standardization is performed to make indicators comparable across different units and orders of magnitude.

For positive indicators, a dimensionless variable $\:{z}_{ij}$ of the *j-*th indicator in the *i*-th region is calculated as

$$\:\begin{array}{c}{z}_{ij}=\frac{{x}_{ij\:}-min\left({x}_{j}\right)\:\:\:\:}{\text{max}\left({x}_{j}\right)-\text{m}\text{i}\text{n}\left({x}_{j}\right)} \end{array}$$.

where min (*x*_*j*_) represents the minimum value and max (*x*_*j*_) denotes the maximum value of the *j*-th indicator.

For negative indicators, the dimensionless variable $\:{z}_{ij}$ of the *i*-th indicator in region *i* is computed as

$$\:\begin{array}{c}{z}_{ij}=\frac{\text{max}\left({x}_{j}\right)\:-{x}_{ij\:}\:\:\:}{\text{max}\left({x}_{j}\right)-\text{m}\text{i}\text{n}\left({x}_{j}\right)} \end{array}$$.

The weight of the calculated indicator is denoted by *p*_*ij*_, where *j* represents the *j-*th indicator and *i* indicates the year *i*.

$$\:\begin{array}{c}{p}_{ij}=\frac{{z}_{ij}}{{\sum\:}_{i=1}^{m}{z}_{ij}} \end{array}$$

Calculate the information entropy, denoted by *e*_*j*_.

$$\:\begin{array}{c}{e}_{j}=-k{\sum\:}_{i=1}^{m}{p}_{ij}\text{ln}\left({p}_{ij}\right) \end{array}$$

Calculate the redundancy of each entropy value, denoted by *d*_*j*_.

$$\:\begin{array}{c}{d}_{j}=1-{e}_{j} \end{array}$$

Calculate the weights for each index, denoted by *w*_*j*_.

$$\:\begin{array}{c}{w}_{j}=\frac{{d}_{j}}{{\sum\:}_{i=1}^{n}{d}_{j}} \end{array}$$

We calculate the index of the development level of the DIE, denoted by *s*_*i*_, for each region in each year.

$$\:\begin{array}{c}{\:\:\:s}_{i}=\sum\:_{j=1}^{n}{w}_{j}{p}_{ij} \end{array}$$

The comprehensive index of the DE, denoted by $\:{s}_{i}$, measures the level of the DE in province *i*. A higher $\:{s}_{i}\:$value indicates a more advanced level of DE, whereas a lower $\:{s}_{i}$ value indicates a less advanced level of DE.

#### Mediating variables and control variables

This study further examines the mediating roles of ISU^42–44^ and ES^30,45^ in the relationship between the DE and the CEE of the logistics industry. In addition, external openness, foreign direct investment, urbanization level, and urban transportation network development are included as control variables.

#### Data sources and descriptive statistics

This study employs panel data from 30 Chinese provinces over the period 2012–2021, excluding Tibet, Hong Kong, Macau, and Taiwan. The data are primarily obtained from the *National Bureau of Statistics of China* and relevant statistical yearbooks, including the *China Statistical Yearbook* and the *China Energy Statistical Yearbook.* A summary of all variables employed in this study is provided in Table 3. Table 4 presents the descriptive statistics of the variables. For missing values in certain years, linear interpolation in Stata to ensure data completeness.

**Table 3:** Variable description.

	Variables	Symbol	Measurement methods	Data source
Independent variable	Digital economy	DE	Entropy method	Calculated by the authors
Dependent variable	Logistics carbon emission efficiency	CEE	Super-SBM	Calculated by the authors
Mediating variable	Industrial structure upgrading	ISU	Gross output of secondary industry as a share of GDP (%)	China Statistical Yearbook
	Energy structure	ES	Ratio of coal consumption to total energy consumption(%)	China Energy Statistical Yearbook
Control variables	Open to the outside world	OPEN	Import/export trade as a percentage of GDP (%)	China Statistical Yearbook
	Foreign direct investment	FDI	The share of the utilization of Foreign Direct Investment (FDI) to the GDP of the province (%)	China Statistical Yearbook
	Urbanization level	URBAN	Urban population as a percentage of total population at year-end in each province (%)	China Statistical Yearbook
	Construction of urban transport networks	TRANS	Based on per capita road space (%)	China Statistical Yearbook

**Table 4:** Descriptive statistics for variables.

Variable	*N*	Mean	Std. dev.	Min	Max	VIF
CEE	300	0.7200	0.4246	0.1592	2.1865	–
DE	300	0.1530	0.1310	0.0081	0.7187	1.61
ISU	300	0.4752	0.2772	0.0177	1.7011	1.28
ES	300	0.9381	0.5353	0.0184	5.2462	1.40
OPEN	300	0.2590	0.2772	0.0075	1.4409	3.14
FDI	300	0.0182	0.0143	0.0001	0.0796	1.39
URBAN	300	0.6023	0.1181	0.3630	0.8960	5.39
TRANS	300	9.3033	0.9093	7.4739	12.7820	4.41

## Results

### CEE (super-SBM)

**Fig. 2:**
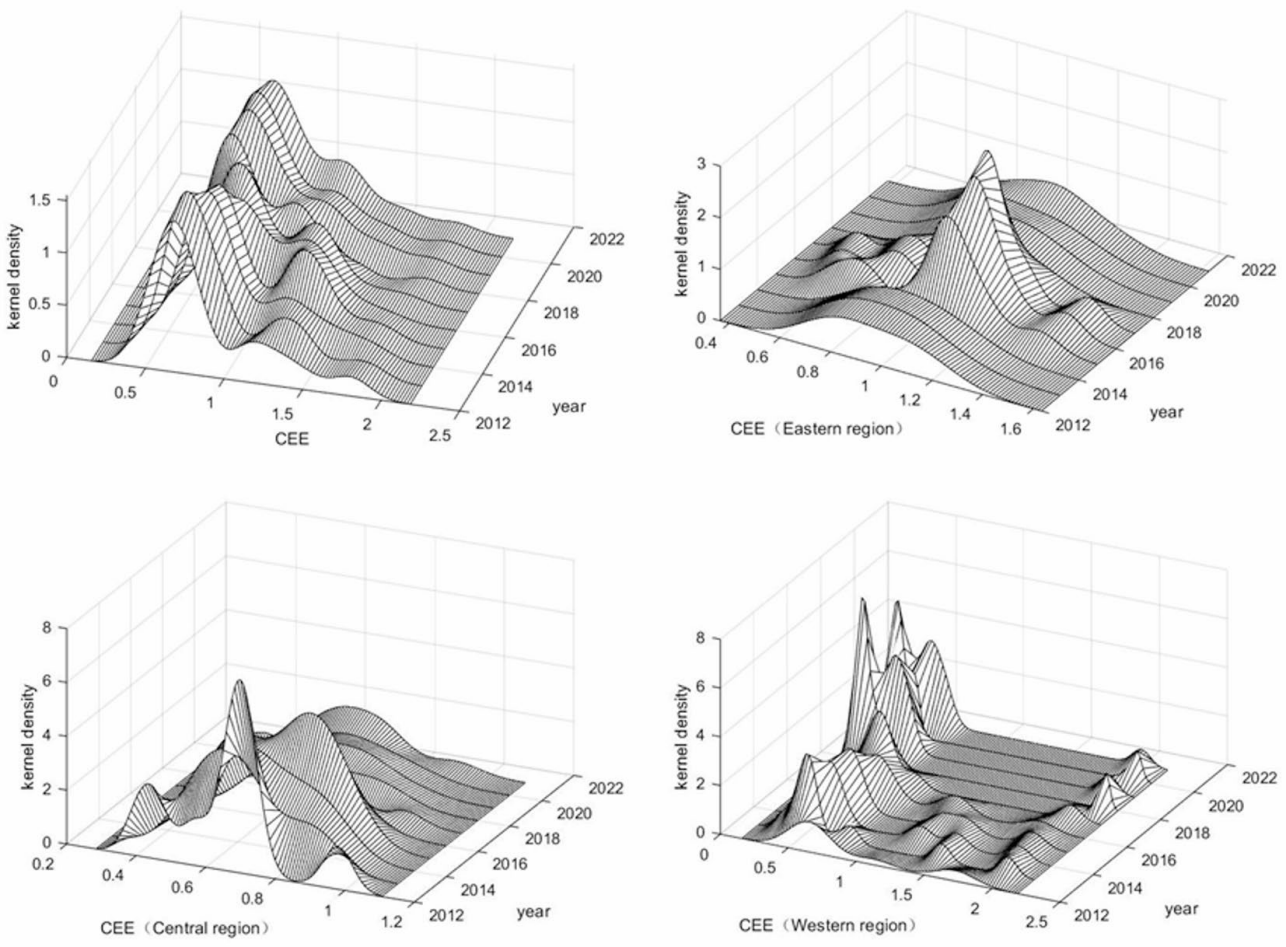
Kernel density curves of the CEE of the logistics industry.

As shown in Fig. 2, the national kernel density curve of China’s logistics CEE from 2012 to 2021 exhibits an overall rightward shift, indicating a continuous improvement in efficiency^46^. At the same time, the persistence of the right tail phenomenon suggests that the CEE gap in the regional logistics industry remains significant. Encouragingly, the gradual shortening of the tail over time reflects a narrowing spatial gap nationwide. At the regional level, the eastern region’s kernel density curve evolved from a unimodal to a multimodal distribution. Then it reverted to a unimodal form, implying that the polarization of logistics CEE first intensified but subsequently eased. The central region exhibits a pattern that is broadly consistent with the national trend. In contrast, the western region presents a more complex distribution, characterized by a dominant peak alongside several secondary peaks. Its overall curve shifts to the left, while the magnitudes of all peaks gradually increase, highlighting substantial internal disparities. The persistent right-tail phenomenon further indicates that, although some provinces in the west maintain relatively high efficiency, the logistics CEE of the region as a whole has declined.

### DE (entropy method)

**Fig. 3:**
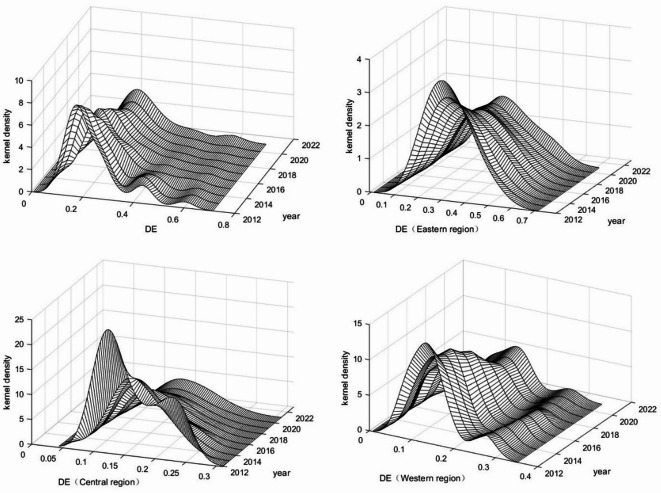
Kernel density curve of the DE.

Figure 3, based on the kernel density estimation method, illustrates the distribution of DE development at the national level as well as across China’s eastern, central, and western regions. Nationally, the kernel density curve of the DE shifts gradually to the right over time, accompanied by a declining peak and a modest widening of the distribution. The flattening of the peak, together with the persistence of a right-tail phenomenon, indicates considerable disparities in DE development across provinces. Nonetheless, the overall rightward shift of the curve suggests steady improvement in DE development throughout the study period^38^. In the eastern region, the curve also exhibits a consistent rightward shift, a declining peak, and broader dispersion, largely mirroring the national trend. Unlike the national distribution, however, the eastern region does not display a right-tail phenomenon, implying that the overall level of DE is relatively high and that no provinces experience severe developmental lag^39^. In the central region, the kernel density curve evolves from a bimodal to unimodal distribution, suggesting that intra-regional disparities are narrowing and gradually converging. By contrast, the western region’s kernel density curve is characterized by one dominant peak alongside a smaller secondary peak. Although the secondary peak is less pronounced, its presence still points to a degree of stratification, reflecting a relatively weak but observable trend of multi-level differentiation in DE development within the western region (Table 5).

### U-shape test

In this study’s model, the linear and quadratic terms of the DE and logistics CEE exhibit statistical associations; however, the evidence is insufficient to independently confirm a nonlinear relationship, which may risk misinterpreting a monotonic relationship as a curvilinear one. To address this issue, we further applied a U-test^45^ for validation. Specifically, first, as mentioned above, the quadratic term of DE is statistically significant and displays the expected sign. Second, Table 6 reports the U-test results, with the upper and lower limits being 0.008 and 0.714 respectively, both of which pass the significance test. Third, the extreme point of the U-shaped curve (0.502) lies within the 95% Fieller confidence interval (0.369, 0.813), and this interval fully covers the observed sample range (0.008, 0.714). Taken together, these results confirmed the existence of a significant inverted U-shaped relationship between DE and logistics CEE, with the inflection point located at 0.502. These findings provide strong evidence for H1, which posits that the DE exerts a nonlinear effect on the CEE of the logistics industry. Specifically, the inverted U-shaped relationship indicates that while improvements in the DE initially enhance logistics CEE, beyond the threshold level of 0.502, further increases in the DE suppress efficiency gains.

**Table 5:** U-test results for the nonlinear relationship between DE and CEE.

U test	Lower bound	Upper bound
Interval	0.008	0.714
Slope	2.471	− 1.062
t-value	3.532	− 1.641
P>|t|	0.000	0.051
Extreme point	0.502	
95% Fieller interval	[0.369; 0.813]	
Overall test	1.64*	

### Mediation effect results

To examine the mediating mechanisms through which DE affects CEE of the logistics industry, this study employed the mediation model discussed earlier. The results are reported in Table 6. Column (1) shows that the impact of DE on ISU is significant at the 1% level, with a positive coefficient for the linear term and a negative coefficient for the quadratic term. This indicates a pronounced inverted U-shaped relationship between DE and ISU, consistent with previous research findings^47^. In the early stages, the application of digital technologies reshapes and optimizes traditional production factors, facilitates the transformation of production methods, and enhances factor allocation efficiency, thereby effectively promoting industrial upgrading and structural optimization^48^. ISU is widely recognized as one of the key pathways for reducing carbon emissions^27^. When the coefficient of the DE’s impact on ISU is negative, it implies a reduction in the share of secondary industry. This structural adjustment guides industrial development in a more balanced direction, reduces the pressure on low-carbon transition, and ultimately enhances carbon efficiency^22^. Column (3) of Table 7 reports the effects of DE on the ES. The linear coefficient is negative, while the quadratic coefficient is positive but not statistically significant, suggesting that the DE can help reduce coal consumption^30^. Energy plays a vital role in sustaining modern economic growth, yet rapid economic expansion has driven strong energy demand. Due to its low cost, coal has long dominated China’s energy consumption structure. However, with the rise of the DE and the accelerated adoption of clean energy, electricity and other green energy sources are gradually replacing coal as the dominant energy supply. After incorporating ISU and ES into the model, columns (2) and (4) of Table 7 show that both variables exert a significant impact on the CEE of the logistics industry. At the same time, the DE still exhibits an inverted U-shaped relationship with the CEE. These findings suggest that the mediating variables play a partial mediating role, indicating that DE can indirectly influence the CEE through ISU and ES.

**Table 6:** Results of the mediating effect model.

Variables	(1)	(2)	(3)	(4)
	ISU	CEE	ES	CEE
DE	1.431*** (0.283)	1.600* (0.818)	-2.213** (0.932)	2.782*** (0.802)
DE^2^	-1.025*** (0.313)	-1.709* (0.880)	1.194 (1.032)	-2.547*** (0.881)
ISU		0.645*** (0.173)		
ES				0.114** (0.053)
Control variable	Yes	Yes	Yes	Yes
Year	Yes	Yes	Yes	Yes
Province	Yes	Yes	Yes	Yes
Constant	-0.236*** (0.151)	1.823*** (0.419)	0.794 (1.388)	2.988** (1.183)
Observations	300	300	300	300
R^2^	0.960	0.871	0.883	0.866

*Robust standard errors are reported in parentheses. ***p < 0.01, **p < 0.05, *p < 0.10.

#### Spillover effects

This study employed the SDM to examine the spatial spillover effects. The results demonstrated that both the direct and spillover effects of the DE on CEE follow an inverted U-shaped relationship, thereby confirming Hypothesis 2. Specifically, the spillover coefficient of the DE was 0.669 and statistically significant at the 10% level, indicating that a 1% increase in the DE level of one region leads to an average 0.669% improvement in the logistics CEE of neighboring regions. Meanwhile, the spillover effect of DE² remained significantly negative, further confirming the existence of spatial nonlinear characteristics. In other words, only when the DE in a region reaches a certain level can it exert a positive spillover effect on CEE of surrounding areas^49^. The observed spillover effects of DE may stem from technological diffusion. Enterprises in regions with higher levels of digitalization often possess advanced low-carbon technologies, and the diffusion of these technologies to surrounding regions helps improve their CEE in the logistics industries^25^. However, as the DE continues to advance and local digital industrialization deepens, local governments may also gradually relocate industries that are difficult to digitally transform to nearby areas. This relocation can increase carbon emissions in surrounding regions, thereby reducing overall CEE^50^.

**Table 7:** Results of spillover effects.

Variable	Direct Effect	Spatial spillover effect
DE	2.534***	0.669*
	(0.721)	(0.373)
DE ^2^	-2.736***	-0.724*
	(0.804)	(0.397)
Control variable	YES	YES
Year	YES	YES
Province	YES	YES

*Robust standard errors are reported in parentheses. ***p < 0.01, **p < 0.05, *p < 0.10.

### Heterogeneity analysis results

China is generally divided into four major economic regions: Eastern, Central, Western, and Northeastern (see Table 8)^51^. This study conducted a heterogeneity analysis based on these regions and their GDP levels^50,52^. The results indicate that the positive effect of the DE on logistics CEE was most pronounced in the Eastern region. This finding can be attributed to the Eastern region’s siginificant advantages in digital talent, technological innovation capacity, and the development level of the digital industry, which together foster more effective digitalization and green transformation. Additionally, we calculated the average annual GDP of each province, derived the median, and used it as a cutoff point to divide the provinces into high and low-economic development groups for group regression analysis. The results show that the impact of the DE on CEE is significant only in regions with higher levels of economic development. This finding is consistent with the view that more advanced economies possess stronger technological capabilities, which facilitate faster digitalization and, in turn, greater improvements in efficiency^14^. By contrast, in less developed regions, the effect of the DE on CEE is not statistically significant (Table 9).

**Table 8:** Regional division of China.

Region	Provinces
Eastern Region (10 provinces)	Beijing, Tianjin, Hebei, Shanghai, Jiangsu, Zhejiang, Fujian, Shandong, Guangdong, Hainan
Central Region (6 provinces)	Shanxi, Anhui, Jiangxi, Henan, Hubei, Hunan
Western Region (11 provinces)	Inner Mongolia, Guangxi, Chongqing, Sichuan, Guizhou, Yunnan, Shaanxi, Gansu, Qinghai, Ningxia, Xinjiang
Northeastern Region (3 provinces)	Liaoning, Jilin, Heilongjiang

**Table 9:** Results of heterogeneity analysis.

	(1)	(2)	(3)	(4)	(5)	(6)
Variable	Eastern Region	Central Region	Western Region	Northeastern Region	GDP(high)	GDP(low)
DIE	2.510** (2.510)	0.966 (2.780)	1.749 (2.521)	8.145 (8.786)	2.164* (1.204)	-1.044 (6.163)
DIE^2^	-2.020* (1.138)	-1.327 (5.155)	-1.171 (5.062)	6.206 (35.330)	-2.569*** (0.986)	11.90 (26.660)
Control variable	Yes	Yes	Yes	Yes	Yes	Yes
Year	Yes	Yes	Yes	Yes	Yes	Yes
Province	Yes	Yes	Yes	Yes	Yes	Yes
Constant	1.816 (1.579)	-1.622 (1.662)	6.618*** (1.914)	12.03** (5.459)	0.081 (1.122)	3.388** (1.379)
Observations	100	60	110	30	150	150
R^2^	0.140	0.724	0.182	0.908	0.155	0.149

*Robust standard errors are reported in parentheses. ***p < 0.01, **p < 0.05, *p < 0.10.

#### Robustness test

To ensure the robustness of the research findings, several verification measures were undertaken. First, the DE was recalculated using the Principal Component Analysis (PCA) method^53^. Second, the ratio of logistics output to carbon dioxide emissions was employed as an alternative indicator of logistics CEE^54^. Third, outliers were removed^55^, and the four municipalities—Beijing, Shanghai, Tianjin, and Chongqing—were excluded from the sample to mitigate the influence of extreme values and non-random factors. Fourth, the study period was shortened^56^, and the model was re-estimated using panel data from 2013 to 2020. Finally, the variables were lagged by one period for additional analysis^57^. The regression analysis shows that the coefficient of the linear term of DE is positive, whereas the quadratic term carries a negative coefficient, which aligns with the results of the baseline regression. This outcome provides additional support for the robustness of the study’s conclusions (Table 10).

**Table 10:** Robustness test results.

	(1)	(2)	(3)	(4)	(5)
DE	0.458*** (0.162)	1.552*** (0.482)	2.758*** (0.896)	2.423*** (0.915)	2.766*** (0.846)
DE^2^	-0.067** (0.029)	-1.714*** (0.539)	-2.376** (1.100)	-2.750*** (0.979)	-2.501*** (0.928)
Control variable	Yes	Yes	Yes	Yes	Yes
Year	Yes	Yes	Yes	Yes	Yes
Province	Yes	Yes	Yes	Yes	Yes
Constant	1.607*** (0.427)	1.363*** (0.259)	3.328*** (0.616)	1.405*** (0.529)	3.657*** (1.329)
R^2^	0.863	0.812	0.846	0.878	0.861

*Robust standard errors are reported in parentheses. ***p < 0.01, **p < 0.05, *p < 0.10.

## Conclusions and implications

This study empirically examines the impact of the DE on the CEE of the logistics industry by utilizing panel data from 30 provinces in China covering the period from 2012 to 2021. First, the DE index was constructed using the entropy method, while the CEE of the logistics industry was measured through the Super-SBM model. Subsequently, Kernel Density Estimation (KDE) was employed to estimate the actual distribution of the two variables and to visually present their patterns, characteristics, and areas of concentration.

Furthermore, to analyze the relationship between DE and CEE, the mediating roles of ISU and ES, as well as the spatial spillover effects and regional heterogeneity, the study sequentially applied a two-way fixed effects model, a mediating effects model, spatial spillover effect, and heterogeneity analysis. Finally, robustness tests were conducted to ensure the reliability of the empirical results.

The main findings are as follows. First, during the study period, both DE and the CEE of the logistics industry improved overall. However, regional disparities remained significant. Second, an inverted U-shaped relationship between DE and CEE was identified, with the turning point estimated at 0.502. Third, ISU and ES were found to play significant mediating roles in the relationship between DE and CEE. Fourth, DE exerted not only direct regional effects but also substantial spatial spillover effects on neighboring provinces, which also exhibited an inverted U-shaped pattern. Finally, heterogeneity analysis revealed that the positive impact of DE on CEE was more pronounced in the eastern and economically developed regions of China.

Based on the above findings, this study offers the following policy implications. First, to ensure the long-term sustainability of the logistics industry, it is essential to continually promote the transition to the DE. To this end, efforts should focus on strengthening key digital infrastructures such as AI, big data, 5G, and IoT, establishing digital logistics pilot zones led by national and local governments, and enhancing digital talent training systems while expanding institutional and policy support. However, the empirical findings of this study indicate that once the DE surpasses a certain threshold, CEE tends to decline. Therefore, the Chinese government should focus on building an optimized monitoring system capable of identifying the maximum threshold at which the DE can exert a positive impact on green growth. By doing so, excessive or redundant investments can be minimized, and the direction and intensity of investment in the DE can be rationally adjusted.

Second, this study demonstrated that the development of the DE can either strengthen or weaken its impact on CEE through the pathways of ISU and ES improvement. Accordingly, the government should not merely expand digital infrastructure but also place greater policy emphasis on guiding industrial structures toward low-carbon and high-efficiency transformation. As evidenced by the case of data centers, the development of the DE may also lead to increased energy consumption. Therefore, from the perspective of ES transition, it is essential to expand the share of renewable energy while simultaneously promoting the use of clean energy within digital infrastructure itself.

Third, this study revealed that the DE exerts a significant inverted U-shaped spatial spillover effect on the CEE in the logistics industry. This suggests that the development of the DE not only affects individual regions but also extends to neighboring areas, improving their CEE up to a certain level while potentially diminishing it once the threshold is exceeded. Therefore, governments should establish data-driven monitoring systems to ensure that the level of DE development in each region remains within the optimal range. In particular, to mitigate regional imbalances, it is necessary to strengthen inter-provincial and inter-city regulatory and cooperative mechanisms, enhance the interconnection of digital infrastructure, and promote the diffusion of green technologies and knowledge. Such regional cooperation and institutional arrangements can help narrow regional disparities and contribute to advancing the low-carbon transition of the logistics industry as a whole.

Fourth, the heterogeneity analysis indicates that the impact of the DE on CEE is more significant in the eastern and high-GDP regions. This highlights the need for differentiated policies tailored to regional development levels rather than a uniform central approach. The eastern and advanced regions can act as leading examples, with their successful experiences institutionalized and transferred to the central and western regions. Meanwhile, less developed regions require strategic investments that combine ISU and ES adjustments rather than simply expanding digital infrastructure. Therefore, the government should adopt differentiated strategies, emphasizing upgrading in advanced regions and capacity-building in less developed areas to reduce regional disparities.

This study makes several contributions. First, it focuses on the logistics industry, a crucial yet underexplored sector in the digital economy transition, and shifts the emphasis of existing research from carbon emission reduction to carbon emission efficiency, thereby providing a more balanced perspective on the sustainability of the logistics sector. Second, it integrates nonlinear dynamics, mediating mechanisms, and spatial spillover effects into a unified framework and incorporates heterogeneity analysis, which together enhance the methodological rigor and robustness of the empirical investigation. Finally, by deriving policy implications from the empirical findings, the study offers practical guidance for promoting sustainable development in the logistics industry.

### Limitations and future research

First, measuring the digital economy remains an imperfect process. Due to limited data availability and the lack of a unified measurement standard, the DE index utilized in this study may not fully capture the diverse differences in digitalization. Therefore, future research should disaggregate the digital economy into specific dimensions to more precisely analyze its heterogeneous impact on CEE. Second, the data used in this study are limited to the provincial level in China. While helpful in understanding overall trends, this approach limits the ability to reflect heterogeneity at the city or firm level, potentially underestimating fluctuations within the logistics industry. Future research could utilize a more granular data set to provide more specific and practical policy implications across multiple dimensions.

## Data Availability

The data used in this study can be available from the corresponding author upon reasonable request.
